# Quantification of Berberine in *Berberis vulgaris* L. Root Extract and Its Curative and Prophylactic Role in Cisplatin-Induced In Vivo Toxicity and In Vitro Cytotoxicity

**DOI:** 10.3390/antiox8060185

**Published:** 2019-06-19

**Authors:** Sarfraz Ahmad, Amina Hussain, Aroosha Hussain, Iskandar Abdullah, Muhammad Sajjad Ali, Matheus Froeyen, Muhammad Usman Mirza

**Affiliations:** 1 Department of Chemistry, Faculty of Science, University of Malaya, Kuala Lumpur 50603, Malaysia; iskandar.a@um.edu.my; 2 Department of Biochemistry, Institute of Molecular Biology and Biotechnology (IMBB), University of Lahore, Lahore 54000, Pakistan; aminahussain796@gmail.com (A.H.); arooshahussain50@gmail.com (A.H.); muhammad.ali@imbb.uol.edu.pk (M.S.A.); 3 Department of Pharmaceutical and Pharmacological Sciences, Rega Institute for Medical Research, Medicinal Chemistry, University of Leuven, B-3000 Leuven, Belgium; mathy.froeyen@kuleuven.be (M.F.); muhammadusman.mirza@kuleuven.be (M.U.M.)

**Keywords:** cisplatin, *Berberis vulgaris*, high-performance liquid chromatography, nephrotoxicity, hepatotoxicity, hyperlipidemia

## Abstract

Cisplatin is amongst the most potent chemotherapeutic drugs with applications in more than 50% of cancer treatments, but dose-dependent side effects limit its usefulness. *Berberis vulgaris* L. (*B. vulgaris*) has a proven role in several therapeutic applications in the traditional medicinal system. High-performance liquid chromatography was used to quantify berberine, a potent alkaloid in the methanolic root extract of *B. vulgaris* (*Bv*RE). Berberine chloride in *Bv*RE was found to be 10.29% *w/w*. To assess the prophylactic and curative protective effects of *Bv*RE on cisplatin-induced nephrotoxicity, hepatotoxicity, and hyperlipidemia, in vivo toxicity trials were carried out on 25 healthy male albino Wistar rats (130–180 g). Both prophylactic and curative trials included a single dose of cisplatin (4 mg/kg, i.p.) and nine doses of *Bv*RE (500 mg/kg/day, orally). An array of marked toxicity effects appeared in response to cisplatin dosage evident by morphological condition, biochemical analysis of serum (urea, creatinine, total protein, alanine transaminase, aspartate transaminase, total cholesterol, and triglyceride), and organ tissue homogenates (malondialdehyde and catalase). Statistically-significant (*p* < 0.05) variations were observed in various parameters. Moreover, histological studies of liver and kidney tissues revealed that the protective effect of *Bv*RE effectively minimized and reversed nephrotoxic, hepatotoxic, and hyperlipidemic effects caused by cisplatin in both prophylactic and curative groups with relatively promising ameliorative effects in the prophylactic regimen. The in vitro cell viability effect of cisplatin, *Bv*RE, and their combination was determined on HeLa cells using the tetrazolium (MTT) assay. MTT clearly corroborated that HeLa cells appeared to be less sensitive to cisplatin and berberine individually, while the combination of both at the same concentrations resulted in growth inhibition of HeLa cells in a remarkable synergistic way. The present study validated the use of *Bv*RE as a protective agent in combination therapy with cisplatin.

## 1. Introduction

Cancer is known to be a major cause of human morbidity and mortality. Different challenges associated with cancer treatment range from late-stage diagnosis to treatment-induced toxicities. Cisplatin (Cis-diamminedichloroplatinum (II)), an alkylating chemotherapeutic drug, has been in clinical practice for over 30 years. It is among the most common frontline medicines for the treatment of some of the most devastating types of solid-organ cancers such as lung, esophagus, head, neck, stomach, testis, and ovary, as well as lymphoma and osteosarcoma [1]. Alongside the benefits of treatment, this iconic drug accompanies a wide range of concurrent nephro-, hepato-, and neuro-toxicities as well as dyslipidemia, which restrict its safe use. Several studies have reported that its cytotoxicity is the major dose-limiting side effect of cisplatin. The etiology concomitant with cisplatin-induced toxicities comprise inflammation, apoptosis, and the generation of reactive oxygen species [1]. These facts suggest that there is an urgent need for therapeutic agents that reduce/ameliorate cisplatin-induced side effects and synergistically improve its chemotherapeutic activity [2,3]. Plants are performing the role of a major health care resource all over the world, and the potential of plant-based remedies has progressively become well known due to their preventive actions and treating human diseases. The immense potential of phytochemicals in pharmaceuticals is evident from the fact that almost one-fourth of the recommended drugs have their origin in phytochemicals [4]. With respect to the treatment of the disorders addressed in this article, the remedial effects of herbs have been well known from many prehistoric civilizations. A number of recent studies have reported a possible role of antioxidants in protecting liver and kidney against cisplatin-induced toxicity. Several plant extracts having antioxidant properties have also been studied as protective agents against toxicities resulting from exposure to cisplatin [5,6].

Barberry is an ancient medicinal shrub known to humans for approximately 2500 years, and it has played a noticeable role in healing [7]. It has been acknowledged in the literature of the medieval era (medical books in Southern Italy). *Berberis vulgaris* L. (*B. vulgaris*) (genus *Berberis*, family Berberidaceae) is a typical garden bush found in Europe, the British Isles, North America, and South Asia, including northern areas of Pakistan. It has long been used for several medical purposes, conspicuously for hepato-protection, as a cardiac tonic, antioxidant, antimicrobial agent, for kidney stones, gall bladder stones, and jaundice [7]. The extract of this plant also reduces blood cholesterol and triglyceride levels, which prevents cardiovascular diseases and maintains normal body functions [8,9]. The major active ingredients found in *B. vulgaris* are isoquinoline alkaloids (berberine, palmatine, jatrorrhizine, berbamine, and oxyacanthine), distributed throughout the plant with the highest concentration in roots. Out of these compounds, berberine, is the most studied and deliberated constituent due to its numerous activities against disorders relating to kidney, liver, heart, inflammation, hyperlipidemia, diabetes, microbes, parasites, reactive oxygen species, and certain cancers [10].

The anticancer activity of *B. vulgaris* along with its hepatoprotective, nephroprotective, and lipid-lowering effects suggest that it could be a potential agent for lowering cisplatin-induced toxicities, as well as synergistically improving its anticancer activity [11]. Based on the previous reports, that different chemopreventive agents can exert synergism with cisplatin in chemo-naive or resistant tumors, we articulated that the combination of berberine and cisplatin might potentiate anti-proliferative activity for the reduction of tumors [12]. In recent years, new approaches based on chemosensitization of cancer cells with reasonably low cancer inhibitors have gained much attention. To validate this hypothesis, we employed the molecular docking procedure to understand the binding affinity of berberine with potential targets. Together with this, we also performed a synergy test for berberine and cisplatin and found a significant enhancement of the anti-proliferative effect as compared with the induction of either agent as a single treatment. We tried to explicate an effective combination chemotherapy that overcomes the high toxicity of conventional chemotherapeutic agents. The MTT (3-(4,5-dimethylthiazol-2-yl)-2,5-diphenyltetrazolium bromide) assay was employed to determine cell viability with respect to whether berberine enhances the growth inhibitory effect of cisplatin in human cervical cancer (HeLa) cells, and the results indicated a remarkable synergic effect of combination therapy. Keeping in view the significance of this important medicinal plant, an evaluation is needed to rationalize the protective effects *B. vulgaris* against the adverse cisplatin-induced effects. This study conducted an in vivo investigation of the curative and prophylactic effects of orally-administrated *B. vulgaris* root extract (*Bv*RE) on cisplatin-induced hepatotoxicity, nephrotoxicity, and hyperlipidemic abnormalities in albino rats [13].

## 2. Results

### 2.1. Molecular Docking Studies

**Figure 1:**
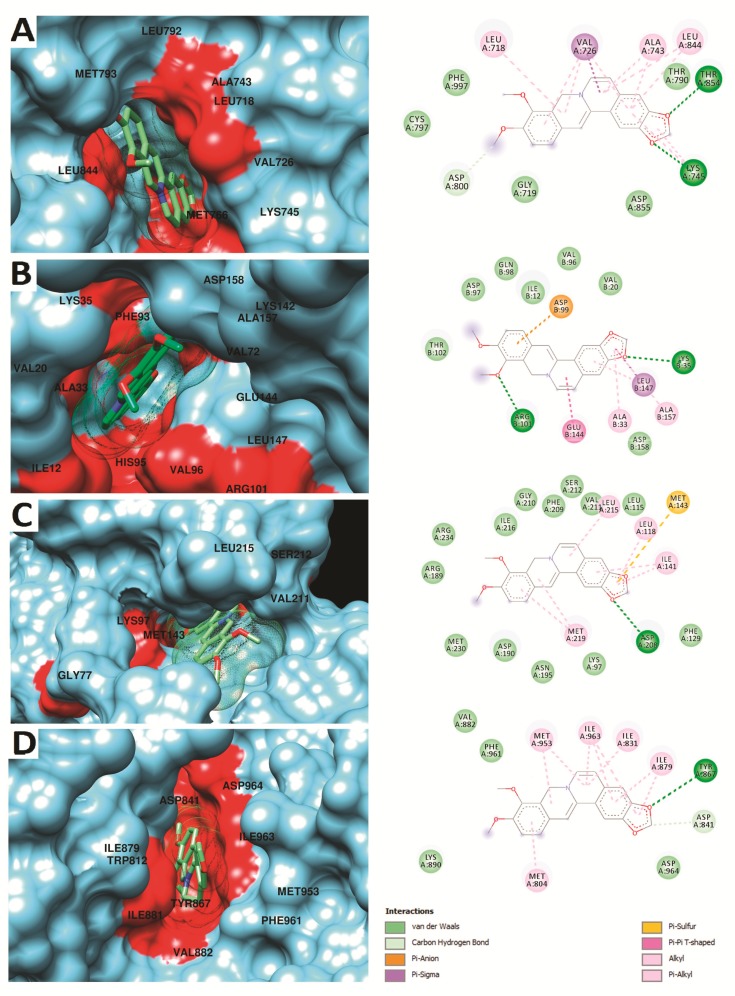
Molecular surface representation of human epidermal growth factor receptor-2 (HER2) (**A**), cyclin-dependent kinase 4 (CDK4) (**B**), mitogen-activated protein kinase kinase (MEK) (**C**), and phosphoinositide 3-kinase (PI3K) (**D**) on the left, while the detailed 2D interactions are illustrated on the right. Berberine with the best docked pose is highlighted in sticks with mesh surface representation inside the binding pocket.

Currently, molecular docking is by far a well-accepted molecular modeling technique, which employs a scoring function to estimate the ligand binding conformation and affinity [14,15,16,17]. Through AutoDock Vina docking, among other target proteins of the mitochondrial apoptotic pathway, berberine had the highest binding energy with phosphoinositide 3-kinase (PI3K) (−10.1 kcal/mol), cyclin-dependent kinase 4 (CDK4) (−9.1 kcal/mol), followed by human epidermal growth factor receptor-2 (HER2) (−9.0 kcal/mol), and mitogen-activated protein kinase kinase (MEK) (−7.3 kcal/mol). Post-docking analysis on the molecular level revealed binding site residues that were found to interact via some promising binding interactions, including hydrogen bonds (H-bonds), van der Waals molecular interactions, and other atomic charge interactions, as illustrated in Figure 1. The docked HER2-berberine complex highlighted the perfect pose inside the active site of HER2 and displayed H-bonds with Thr854 and Lys745. Along with this, some key residues interacted through van der Waals interactions (Figure 1A). However, the docked CDK4-berberine complex, Lys35, and Arg101 participate in H-bonds with berberine and showed some other interactions; including van der Waals, π-sigma, carbon-hydrogen, and π-anion interactions (Figure 1B). Likewise, the docked MEK-berberine and PI3K-berberine complex established only one H-bond with Asp208 (in MEK) and Tyr867 (in PI3K). In both complexes, berberine displayed a network of molecular interactions (van der Waals, Pi–Pi, and π-sigma bonds) with the main active site residues of MEK and PI3K (Figure 1C,D). It was further examined that the pyrrole ring of berberine was actively participating in H-bonds with all four representative proteins and found deep inside the groove, while the dimethoxy aromatic ring of berberine was well fitted in the hydrophobic groove of all four proteins, which suggested the existence of strong π-sigma, π-anion, Pi–Pi stacking, and certain electrostatic interactions, which confirm the inhibitory effect of berberine through these key proteins of the mitochondrial apoptotic pathway.

### 2.2. MTT Assay

**Figure 2:**
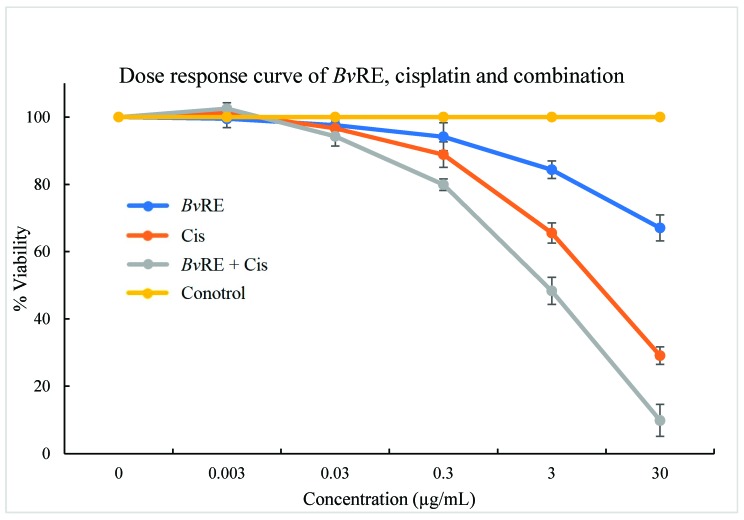
MTT assay dose-response curve of *Berberis vulgaris* root extract (*Bv*RE), cisplatin, and their combination on HeLa cells.

HeLa cells were treated with both *Bv*RE and cisplatin at concentrations of 30, 3, 0.3, 0.03, and 0.003 µg/mL, individually and in combination, to examine the combined effect of *Bv*RE and cisplatin. The cell viability was observed to be decreased with the increase in concentration. At the maximum tested concentration (30 µg/mL), *Bv*RE, cisplatin, and *Bv*RE-cisplatin showed 67.06%, 29.11%, and 9.86% viability, respectively. These results confirmed the additive effect of the combination therapy (Figure 2).

### 2.3. Quantification of Berberine in BvRE

**Figure 3:**
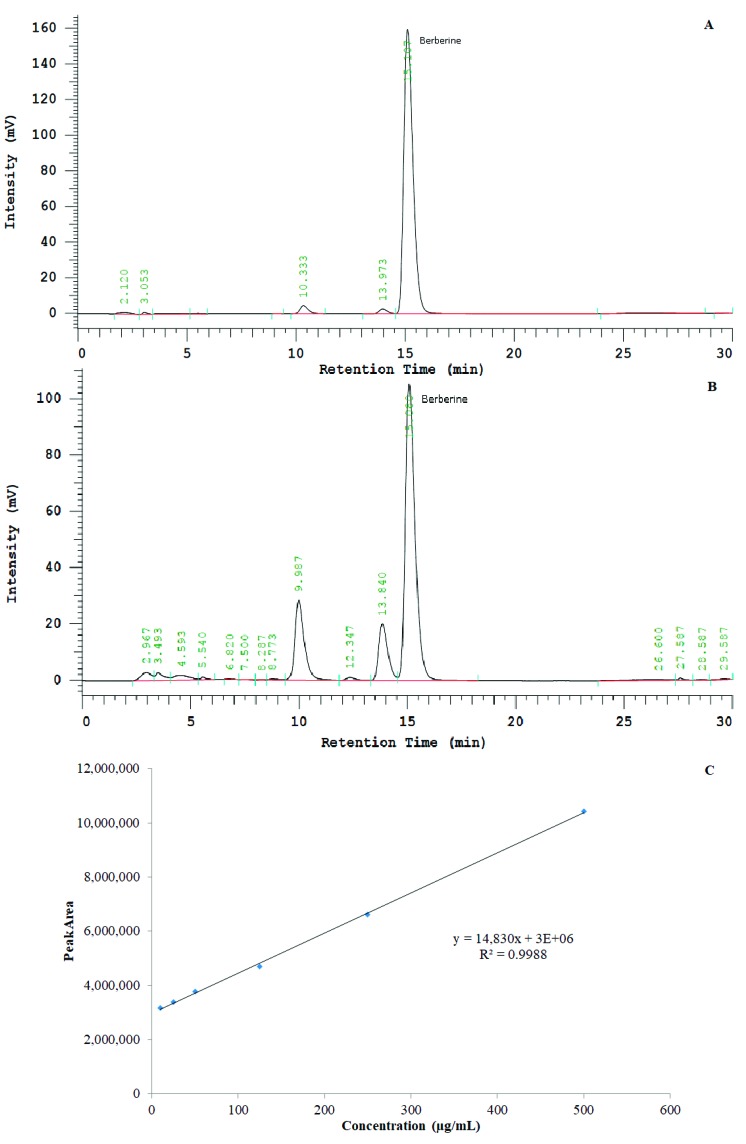
(**A**) HPLC chromatograph of standard berberine chloride at a 125-µg/mL concentration. (**B**) HPLC chromatograph of *Berberis vulgaris* root extract (*Bv*RE) at a 1-mg/mL concentration. (**C**) Standard curve of berberine chloride, concentration (µg/mL) against area under the peak.

Chromatographs of standard berberine chloride and test *Bv*RE solutions are shown in Figure 3. A standard curve with *R*^2^ = 0.9988 was achieved by plotting berberine peak areas from standard solutions against the respective concentrations (Figure 3). The berberine peak area of the test *Bv*RE was 3,305,202 (area is in relative response units with minutes along x-axis and millivolts along y-axis in chromatogram), corresponding to 20.58 µg/mL of berberine chloride. The concentration of the *Bv*RE test solution was 200 µg/mL. Thus, 200 µg of extract contained 20.58 µg of berberine chloride (10.29% *w/w*). The yield of extract from the dry roots of *B. vulgaris* was 23.67%. Hence, the amount of berberine chloride in dry root mass was 2.44% *w/w*. According to these observations, the dose of 500 mg/kg/day *Bv*RE was equivalent to 51.45 mg/kg/day of berberine chloride.

### 2.4. Effect of BvRE on Cisplatin-Induced Hepatotoxicity

Both groups with cisplatin-induced toxicity, i.e., prophylactic control and curative control groups, showed a significant increase of serum alanine transaminase (ALT), serum aspartate aminotransferase (AST), tissue malondialdehyde (MDA), and a decrease of tissue catalase (CAT) levels as compared to the normal control group at *p* < 0.05, depicting marked induction of liver dysfunction. In prophylactic and curative groups, the results indicate that the ameliorative effects of *Bv*RE significantly reversed the parameters close to the normal control. These restorative effects were more pronounced in the prophylactic regimen in comparison with the curative regimen (Table 1).

**Figure 4:**
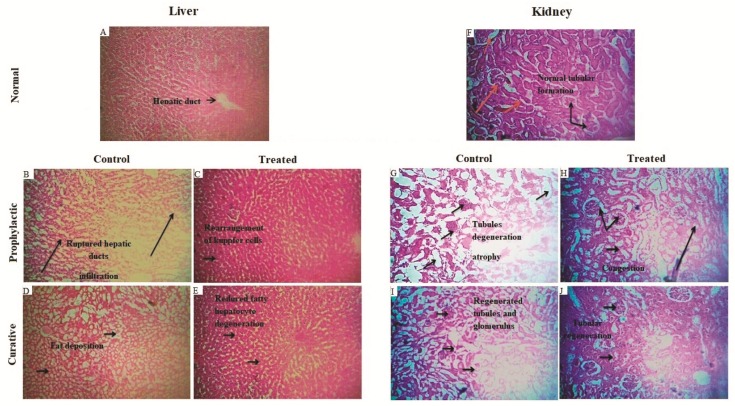
Histopathology of rat liver sections: (**A**) Normal control group: no significant deviation from the normal histological architecture of hepatocytes was observed. The arrowhead represents the position of the central vein. (**B**) Prophylactic control group: cisplatin-induced toxicity resulted in significant devastation of hepatocytes with the disarray of the architecture as compared to the normal control group. (**C**) Prophylactic group: initiation of significant regenerative changes and more regular alignment of hepatocytes as compared to the prophylactic control group. (**D**) Curative control group: revealed significant fatty changes and degenerative changes in the architecture of damaged hepatocytes as compared to the control group. (**E**) Curative group: exhibited regenerative changes in the hepatocyte architecture as compared to the curative control group. Histopathology of rat kidney sections: (**F**) Normal control: no significant deviation from the normal architecture of glomerulus and tubules. The portions marked by the arrowhead indicate glomerulus and tubular portions of nephrons. (**G**) Prophylactic control group: the normal architecture was significantly affected by the toxicity of cisplatin representing marked Glomeruli congestion, atrophy, and tubular damage. (**H**) Prophylactic group: revealed initiation of marked regenerative changes in the architecture through glomerular and tubular regeneration as compared to the prophylactic control group. (**I**) Curative control group: significant congestion and degeneration of glomeruli and marked destruction of tubules were observed as compared to the normal control group. (**J**) Curative group: initiation of regenerative changes in the architecture (glomerular and tubular regeneration) was observed as compared to the curative control group.

Moreover, these findings were supported by the histological studies of the liver, which confirmed the manifestation of tissue damage induced by cisplatin as compared to the normal control group and the reversal of the tissue structure using *Bv*RE (Figure 4A–E). No prominent divergence from the normal histological architecture of hepatocytes was observed in the photomicrograph of the normal control group (Figure 4A, where the arrowhead represents the position of the central vein). The administration of cisplatin in the prophylactic control group (Figure 4B) resulted in significant devastation of hepatocytes with the disarray of the architecture as compared to the normal control group. The prophylactic administration of *Bv*RE in the prophylactic group (Figure 4C) presented significant regenerative changes and more regular alignment of hepatocytes close to the normal control group (Figure 4A) as compared to the prophylactic control group (Figure 4B). The curative control group (Figure 4D) revealed significant fatty and degenerative changes in the architecture as a result of damaged hepatocytes as compared to the control group. The administration of *Bv*RE in the curative group (Figure 4E) after cisplatin-induced damage exhibited quick regenerative changes in the hepatocyte architecture as compared to the curative control group (Figure 4D).

### 2.5. Effect of BvRE on Cisplatin-Induced Nephrotoxicity

The striking cisplatin-induced nephropathy in the prophylactic and curative control groups was exhibited by increased levels of serum urea, serum creatinine, and tissue MDA and reduced levels of serum total protein and tissue CAT as compared to the control group at *p* < 0.05. In the prophylactic and curative groups, the nephroprotective activities of *Bv*RE effectively minimized the toxicity induced by cisplatin with a marked improvement in the prophylactic regimen. Blood urea level was the only parameter that was more disturbed by the presence of *Bv*RE (Table 2).

A piece of strong evidence in support of the above-mentioned results was provided by histopathological studies of the kidney sections (Figure 4F–J). Figure 4F shows the normal histological architecture of nephrocytes in the normal control group depicting the normal architecture of glomerulus and tubules. The regions labeled with the arrowhead indicate glomerulus and tubular parts of normally-functioning nephrons. Figure 4G shows considerable destruction of nephrocytes with dilation in Bowman’s space and epithelial desquamation in the prophylactic control group. The normal architecture was significantly affected by the toxicity of cisplatin, representing marked glomeruli congestion, blood vessel congestion, atrophy, and tubular damage. The histopathological investigation of the prophylactic group (Figure 4H) revealed an insignificant deviation from the normal nephrocyte structure in terms of glomerular and tubular integrity as compared to the prophylactic control group. In the curative control group (Figure 4I), the architecture was comparatively similar to the prophylactic control group (Figure 4G) with significant congestion and degeneration of glomeruli and marked destruction of tubules as compared to the normal control group (Figure 4F). In the curative group (Figure 4J), initiation of marked degenerative changes in the architecture through glomerular and tubular regeneration was shown as compared to the curative control group (Figure 4I).

### 2.6. Effect of BvRE on Cisplatin-Induced Dyslipidemia

Increased total cholesterol (TC) and triglycerides level (TG) of the prophylactic and curative control groups showed cisplatin-induced dyslipidemia at *p* < 0.05. However, the prophylactic and curative groups showed that the presence of *Bv*RE rendered promising results in restoring normal levels of TG and TC (Table 3).

## 3. Discussion

Berberine is a bona fide principal constituent of *Bv*RE. Berberine chloride has an experimental log *p*-value of −1.5 and an aqueous solubility of 8.50 ± 0.40 mM at physiological temperature, suggesting its fairly hydrophilic nature [18]. The oral bioavailability of berberine is less than 1% in rats, while its closely analogous isoquinoline alkaloids, palmatine and jatrorrhizine, significantly present in *Bv*RE, have 10.98% and 43.90% bioavailabilities, respectively [19]. Berberine is absorbed into the intestine after conversion into dihydroberberine (having intestinal absorption five-fold higher than berberine) via microbial flora, probably nitroreductases, in the digestive system. The dihydroberberine is converted back to berberine in the intestinal tissues through oxidation. In spite of low bioavailability, the very low plasma concentration of berberine is maintained for 36 h, and it is widely distributed in liver, kidney, and fat. In rats, the LD_50_ value of *Bv*RE administrated through the oral route is 1280 mg/kg [20]. According to Section 2.3, HPLC-based quantification showed that the dose of 500 mg/kg/day *Bv*RE was equivalent to 51.45 mg/kg/day of berberine chloride. Thus, the selected dose of 500 mg/kg/day for eight days was considered to be well tolerated.

A large number of drug leads were ruled out only due to their acute and chronic toxicities. The prevalence of drug-induced toxicities is always an ongoing concern, which stays in focus during the drug development process and clinical practice. We tried to explicate an effective chemotherapy combination that overcomes the high toxicity of conventional chemotherapeutic agents, i.e., induced toxicity of cisplatin. In recent years, new approaches based on chemo-sensitization of cancer cells with reasonably low cancer inhibitors have gained much attention. The present study focuses on the synergic effect of a toxic agent (cisplatin) in combination with a non-toxic cancer inhibitor (berberine) accompanying the efficacy of the treatment and combination decreasing tumor growth significantly compared to single-agent administration. The most momentous events of toxicities appear during the administration of anticancer drugs particularly affecting kidneys, liver, and heart. Although cisplatin is the queen of the chemotherapeutic drugs among Food and Drug Administration (FDA)-approved drugs with applications in numerous cancers, it is reported to be a nephro- and hepato-toxic drug. The cytotoxic effect of cisplatin is enhanced by an increase in the dosage quantity, but at higher doses, severe damage occurs in kidneys, peripheral nerves, liver, and other tissues. Therefore, the use of cisplatin as a chemotherapeutic agent came into question in early clinical trials [21,22]. A continuous search is in progress for therapeutic agents that endow nephroprotection against cisplatin and other platinum drugs. In recent times, the focus on plant research has increased all over the world, and a large body of evidence suggests that plants are immense and valuable reservoirs of unique remedial agents, having a remarkable structural diversity among their active components [7,23]. In the present study, serum, urea, creatinine, total protein, ALT, AST, and lipid profile (TC and TG), along with analysis of tissue lipid peroxidation (MDA), antioxidant status (CAT), and histopathological examination of both liver and kidney were performed by keeping in view the antioxidant role of *Bv*RE on cisplatin-induced toxicities (hepato- and nephro-toxicity) and dyslipidemia [24].

Various studies demonstrated that cisplatin-induced hepatotoxicity through membrane rigidness, oxidative damage, lipid peroxidation, and interaction with protein sulfhydryl groups [25]. This results in increased ALT and AST levels, indicating damage of the cell membrane and leakage of intracellular constituents of hepatocytes [26]. Thus, serum transaminases ALT and AST are biomarkers for liver fitness. In prophylactic and curative control groups, ALT was 109 and 102.8 U/L respectively, which is more than a three-fold increment over the normal control group (34 U/L). In prophylactic, curative, and normal control groups, serum AST levels were 76.80, 77.20, and 40.60 U/L, respectively, i.e., nearly a two-fold elevation in prophylactic and curative control groups as compared to the normal control group. These results exhibit the existence of liver damage caused by cisplatin. The groups treated with *Bv*RE in a prophylactic and curative manner in the presence of cisplatin presented serum ALT and AST values quite close to the normal control group except the serum AST level in the curative group (61 U/L), which was about 1.5-times higher than the normal control group (Table 1).

The level of MDA in liver tissues showed the extent of oxidative stress. It is a product of oxidative degradation of lipids by a free radical chain reaction, which initiates with reactive oxygen species (ROS) like hydroxyl or hydroperoxyl free radicals, resulting in the damage of cell membrane. An increased liver tissue MDA level in prophylactic and curative control groups (38.5 and 31.5 nmol/10 mg, respectively) as compared to the normal control group (22.5 nmol/10mg) shows considerable lipid decomposition and cell damage, resulting in elevated toxicity. Prophylactic and curative groups showed a considerable decrease in MDA level (16.5 and 18.8 nmol/10 mg, respectively), resulting due to the antioxidant activity of *Bv*RE. The effect was more pronounced in the prophylactic group as compared to the curative group because the already present antioxidants of *Bv*RE scavenged free radicals generated by cisplatin (Table 1). CAT is an enzyme that catalyzes the decomposition of hydrogen peroxide into water and oxygen, thereby defending cells from oxidative degradation from reactive oxygen species. Its amount in liver tissues of the normal control group was 25.98 U/mg. Inactivation of CAT in prophylactic and curative control groups (14.84 and 17.18 U/mg, respectively) showed the generation of ROS by cisplatin and hepatic fibrosis. In the prophylactic drug regime, antioxidants of *Bv*RE played their role against the ROS of cisplatin and maintained the CAT level close to the normal value (25.68 U/mg). This effect was not promising in the curative group, where the level only raised to 18.78 U/mg (Table 1).

Cisplatin is eliminated from the kidney through both tubular secretion and glomerular filtration. Its concentration in kidney rises as compared to blood. This active accumulation of cisplatin by renal parenchymal cells results in DNA damage, activation of cell death and survival pathways, generation of ROS, and inflammatory reaction, resulting in nephrotoxicity [27]. The DNA binding, antioxidant, and anti-inflammatory activities of berberine in *Bv*RE could result in a protective effect against cisplatin-induced damage in kidney [28]. In the current study, varied renal parameters after cisplatin treatment indicated the nephrotoxic effects of cisplatin. Kidney tissue MDA level was increased about four- and 2.7-fold in prophylactic and curative control groups, respectively, as compared to the normal control group, which shows a very high prevalence of renal damage by ROS. On the other hand, in *Bv*RE-treated groups, the MDA level was 2.1- and 1.6-fold higher in prophylactic and curative groups, respectively, as compared to the normal control group, showing a considerable decrease in lipid peroxidation as compared to the respective control groups (Table 2); whereas, the CAT level of the normal control group (20.44 U/L) was lowered nearly to five- and 2.9-fold in prophylactic and curative control groups (3.97 and 7.09 U/L, respectively), while prophylactic and curative groups had an increased CAT level with values of 11.18 and 12.36 U/L. This shows that *Bv*RE tends to restore the normal redox balance of the liver (Table 2). Increased serum creatinine and decreased urea and TP in prophylactic and curative control groups showed remarkable glomerulonephritis or nephrotic disorder. The findings are correlated with previous studies [29,30]. The nephroprotective activities of *Bv*RE efficiently reduced the toxicity in prophylactic and curative groups.

Elevated total cholesterol (TC) and triglyceride (TG) level portray cisplatin-induced dyslipidemia [31]. Cisplatin disturbed the metabolic pathway of lipid metabolism including TC and TG in prophylactic and curative control groups as compared to the other groups of the current study. The accumulation of lipid contents in the blood vessels, as well as in renal tubules is also probable. Studies have reported two different points regarding dyslipidemia, including hypercholesterolemia and hyperglyceridemia. An elevated level (41%) of cholesterol was reported in the patients receiving cisplatin who had no previous history of hypercholesterolemia [32]. Moreover, dyslipidemia (hypercholesterolemia and hyperglyceridemia) has been observed as the adverse effects of cisplatin-based chemotherapy [33]. It was reported previously that berberine is a promising upregulator of the LDL receptor, and it can produce about a 44 and 35%, respectively, reduction in serum total cholesterol and triglyceride levels in hamsters [34]. In the present study, *Bv*RE lowered the TC level of prophylactic and curative groups quite close to the normal control group (Table 3). A decrease of 35 and 23% in TC levels was found in prophylactic and curative groups, respectively, as compared to their respective control groups. Serum TG level was also lowered in prophylactic and curative groups as compared to the elevated value in their respective control groups, but it was not in close agreement with the normal control group. The cytotoxic effect of cisplatin and berberine alone and in combination can be seen in Figure 4. The results showed that berberine alone did not produce remarkable effects on cell lines after 48 h. Therapeutic efficacy of cisplatin is not fully known; however its cytotoxic action against tumor cells is thought to be mediated through the formation of DNA-cisplatin adducts that lead to inhibition of transcription and/or DNA replication [35]. Moreover, its cytotoxic effect is deemed to be due to the generation of reactive oxygen species through oxidative stress-dependent mechanisms [36]. Cisplatin has been employed for the treatment of various tumors, but its side effects are not ignorable [37]. It has been reported that cisplatin induces its cytotoxic effect via the MAPK pathway and stress-mediated activating transcription factor 3 (ATF3) [38,39]. Resistance towards cisplatin in cancer cells may imply various explanations including the increased repair capacity of DNA, tolerance to DNA damage, decreased drug accumulation, as well as enhanced inactivation of drug [40].

Moreover, berberine shows its anti-cancer effects through multiple targets. It can cause cell arrest in multiple human cancer cell lines [41]. MTT was employed to determine cell viability with respect to whether berberine enhances the growth inhibitory effect of cisplatin in HeLa cells. IC_50_ doses of combination chemotherapy were added to the untreated cells or pre-treated for 48 h with the IC_50_ dose of cisplatin. It suppresses the expression of antiapoptotic proteins in cancer cells and downregulates telomerase activity [42]. It also obstructs the synthesis of DNA in ovarian cancer cells by inhibiting two enzymes in the pathway, thymidylate synthase and dihydrofolate reductase [43]. Various studies have reported the antioxidant action of berberine [28,44,45]. Berberine and its derivatives upregulate catalase and superoxide dismutase expression, in a dose-dependent manner, in human fibrosarcoma cells (HT1080) [46]. These findings, along with molecular docking of berberine suggest that dual targeting of the mitochondrial apoptotic pathway (HER2/PI3K/MAPK) combined with cisplatin can be a favorable strategy to enhance the antiproliferative effect by reducing cytotoxicity. Furthermore, the putative mechanism of these results relies on the synergistic effect of *Bv*RE and cisplatin in cancer cells and the side effect amelioration in the normal cells.

## 4. Materials and Methods

### 4.1. Animals

Albino rats were obtained from the animal house of The University of Lahore, Pakistan, and trials performed were approved by the Animal Ethics Committee of the University (The approval code is "IMBB-12-0051"). Adult healthy male albino rats weighing between 180 and 200 g were used in the present study. Animals were housed in standard cages where they were provided with free access to water and a standard diet under controlled conditions of a temperature of 25 ± 2 °C and a normal photoperiod (12 h dark and light) throughout the rearing and dosing period.

### 4.2. Drugs and Chemicals

Standard cisplatin was purchased from Bio Vision, Inc. (Milpitas, CA, USA). The biochemical analyses were carried out by using the kits from Human Diagnostics (Wiesbaden, Germany). Standard berberine chloride was purchased from Alfa Aesar (Karlsruhe, Germany). All other reagents/chemicals were of analytical grade and thus used without further purification.

### 4.3. Preparation of Plant Extracts

*B. vulgaris* roots were collected from Swat valley in northern areas of Pakistan and characterized and identified by Dr. Muhammad Sajjad Ali (Assistant Professor, Plant Biotechnology, The University of Lahore, Pakistan). A voucher specimen (No. 03048) was submitted to the herbarium of The University of Lahore. The roots were thoroughly washed with water at ambient temperature, subjected to air drying in the shade, pulverized, and sieved through 80 mesh sieves. The 100 g of powder were subjected to extraction by macerating in 500 mL of 70% methanol in water in a well capped glass jar, kept at 25 °C in an orbital shaker at 100 rpm, away from direct sunlight. After 24 h, the liquid was filtered, the residue washed three times with 100 mL 70% methanol, and the filtrates combined and concentrated on the rotary evaporator at 40 °C and lyophilized to obtain 23.67 g (yield = 23.67%) dry *Bv*RE.

### 4.4. Molecular Docking Studies

It has been well reported that cisplatin acts through DNA adducts and activates certain pathways, including important mitochondrial apoptotic pathways [47]. For the synergistic effect of cisplatin in combination with berberine, it was important to find out the potential targets of berberine that are involved in proliferation and angiogenesis. To do this, an extensive literature survey was performed that identified the potential molecular targets for berberine. Several acceptable molecular mechanisms exist that explain the anti-proliferative effects of berberine. The antiproliferative ability of berberine is evident from various in vitro studies on human cancer, in which it can cause cell cycle arrest and induced apoptosis [41,48]. One of the main pathways berberine employs for its antiproliferative effects is the induction of the mitochondrial apoptotic pathway and the HER2/PI3K/MAPK pathway, which induces G1 arrest and apoptosis via regulation of the Cdk-cyclin cascade [41,49]. Based on these observations, we carefully selected four potential targets for molecular docking studies of berberine. These included: CDK4 (PDB ID: 2W96, resolution: 2.30 Å) [50], HER2 (PDB ID: 3POZ [51], resolution: 1.50 Å), MEK (PDB ID: 4U81, resolution: 2.70 Å) [52], PI3K (PDB ID: 4HVB, resolution: 2.35 Å) [53], and their three-dimensional structures were retrieved from the research Collaboratory For Structural Bioinformatics (RCSB), Protein Data Bank (PBD). Prior to molecular docking, the representative protein structures were minimized and optimized using the same protocol as described elsewhere [14,15,54,55]. The prepared protein structures were then used for molecular docking using AutoDock Vina (Scripps Research, La Jolla, CA, USA) [56]. The structure of the chemotherapeutic agent berberine was retrieved from ChemSpider [57] and prepared for docking using Chimera (RBVI, University of California, San Francisco, CA, USA).

### 4.5. MTT Assay

HeLa cells (HeLa ATCC^®^ CCL-2^TM^) were provided by the Center of Research in Molecular Medicine, The University of Lahore, Lahore Pakistan. The cells were harvested at 80% confluence, by treating a medium-free monolayer with trypsin EDTA. Cell counts were performed with a hemocytometer, and cell viability was determined with trypan blue. The cell suspension was adjusted to a final concentration of 7 × 10^5^ cells/mL in DMEM with 10% Fetal Calf Serum (FCS). Cells were dispensed to tissue culture-grade 96-well plates, with each microwell receiving 100 µL, i.e., 7000 cells/well. The plate was incubated at 37 °C for 12 h in a 5% CO_2_ incubator. Stock solutions (1 mg/mL) of test *Bv*RE and cisplatin were prepared in deionized water, and test concentrations (0.006, 0.06, 0.6, 6, and 60 µg/mL) of *Bv*RE and cisplatin were prepared by diluting stocks in growth media. In order to investigate the synergistic effect, the dilutions having mixtures of *Bv*RE and cisplatin both at the same concentrations as mentioned above were prepared in a similar manner. Test drug dilutions (100 µL) were applied giving a final well volume 200 µL and concentrations of 0.003, 0.03, 0.3, 3, and 30 µg/mL. After 48 h, the MTT (3-[4,5-dimethylthiazol-2-yl]-2, 5-diphenyltetrazolium bromide) reagent prepared in PBS at a rate of 5 mg/mL was applied 25 µL/microwell, followed by incubation for 5 h. The entire content of each microwell was carefully pipetted out without disturbing the cells at the well floor. In order to solubilize the formazan crystals, 100 µL of dimethyl sulfoxide was dispensed to each well. The absorbance was recorded with the help of a plate reader at a wavelength of 570 nm. Negative control wells received all reagents except any drug. The percentage viability was calculated, and dose-response curves were plotted using percentage viability against concentration (µg/mL). Results represent the mean ± S.D. of six readings, three determinations of two independent experiments [58].

### 4.6. Quantification of Berberine in BvRE

The Hitachi Primaide HPLC system (Hitachi High-Technologies Cooperation, Tokyo 105-8717, Japan) equipped with a Primaide 1110 pump, Primaide 1310 column oven, Primaide 1410 UV/Vis detector, Primaide 20-µL sample loop, and Primaide System Manager data acquisition software (Version 1.0) were used. While, Idex 7725i analytical front-loading injection valve with manual sampler (Idex Health & Science, Saitama 332-0035, Japan), 50-µL Hamilton syringe (Hamilton Company Japan K.K. Tokyo 105-6031, Japan), and CNW Athena C-18-WP column (5 µm, 100 Å, 4.6 × 250 mm) (CNW Technologies GmbH, Düsseldorf, Germany) were used for quantification of berberine in *Bv*RE. Elution was achieved isocratically with 50% acetonitrile and 50% water containing 3.4 gm/L sodium dodecyl sulfate and 6 gm/L sodium dihydrogen phosphate with a flow rate of 1 mL/min and the column oven at 40 ± 1 °C, and the absorbance of berberine was measured at 345 nm [59]. Standard berberine chloride was accurately weighed (on a weight balance with a detection limit of ±0.1 mg) and dissolved in methanol to get a stock solution of 1000 µg/mL. This solution was used to prepare further dilutions of 500, 250, 125, 50, 25, and 10 µg/mL. The test solution of *Bv*RE was prepared at a concentration of 200 µg/mL. During each run, 10 µL of the standard or test solution were used. The experiment was repeated in triplicate, and the average berberine peak area for each solution was calculated. Peak area was plotted against concentration to get the standard curve, and the amount of berberine in the test *Bv*RE solution was calculated [59].

### 4.7. Experimental Design

The U.K.’s Animals (Scientific Procedures) Act, 1986, and associated guidelines were observed, and the animals were divided into five groups having five rats in each group (*n* = 5). The doses of cisplatin (2 mg/L) and *Bv*RE (500 mg/mL) were prepared in water for injection. A single high dose of cisplatin (4 mg/kg body weight) was injected via intra-peritoneal route, while nine doses of *Bv*RE (500 mg/kg body weight) were administered orally through gastric intubation at 24-h intervals. The groups were treated according to the study plan described in Table 4 [13].

### 4.8. Biochemical Analysis

Blood was collected from each animal by heart puncture under chloroform anesthesia, and serum was separated for biochemical analysis.

#### 4.8.1. Preparation of Tissue Homogenate

The 10% (*w/v*) liver and kidney tissue homogenates were prepared in 10-mM phosphate buffer (pH 7.4). The homogenate was centrifuged at 13,000 rpm for 10 min at 4 °C. Tissue supernatant samples were separated and used for the estimation of oxidative stress status [30].

#### 4.8.2. Determination of Renal and Liver Function

Serum aspartate aminotransferase (AST), alanine aminotransferase (ALT) activities, serum total cholesterol (TC), serum triglyceride (TG), serum urea, serum creatinine, and serum total protein (TP) were determined by the kit method [60,61,62,63,64].

#### 4.8.3. Estimation of Oxidative Status

Catalase (CAT) activity was measured by the method of Aebi [65], and lipid peroxidation level was assessed as malondialdehyde (MDA, thiobarbituric acid reactive substances) by the method of Ohkawa [66].

### 4.9. Histopathological Examination

Small portions of liver and kidney tissues were collected and fixed in 10% formalin until histopathology analysis was performed. The tissues were embedded in paraffin wax blocks, and 5 μm-thick tissue sections were then sequentially stained with hematoxylin-eosin [13].

### 4.10. Statistical Analysis

The values from each group are presented as the mean of five readings (*n* = 5) and their standard deviation (mean ± S.D.). In order to evaluate the statistical significance and individual comparisons, the obtained data were subjected to one-way ANOVA followed by a post-hoc Scheffé test on SPSS (ver. 16, IBM Corporation, Armonk, NY, USA). Values were considered as statistically significant when significance was less than 0.05 (*p* < 0.05) [67].

## 5. Conclusions

The present study endorsed and substantiated the historical use of *B. vulgaris* as a curative agent for the treatment of kidney, liver, and heart disorders. It was concluded from the consequences of the present study that both prophylactic and curative regimens of *Bv*RE (at a dose of 500 mg/kg/day equivalent to 51.45 mg/kg/day berberine chloride) had a marked protective activity against cisplatin-induced nephrotoxicity, hepatotoxicity, and hyperlipidemia in rats. The present research work corroborates and dignifies the conventional beliefs on *B. vulgaris* as a multi-targeting therapeutic agent. The pre- and post-treatment with *Bv*RE (500 mg/kg) displayed a marked protection against the impaired liver and kidney function, as well as dyslipidemia induced by cisplatin. Both treatments (prophylactic and curative) reversed the effects of cisplatin-induced impairments, whereas the prophylactic course of therapy revealed comparatively better results as compared with the curative course of therapy against disorders induced in both liver and kidney and lipid levels. This effect proves that that pre-existence of the protective agents of *Bv*RE effectively detoxifies the toxic effects of cisplatin on liver and renal function markers, lipid profile, lipid peroxidation, and antioxidant status, which appear to accelerate normal redox homeostasis at the cell level. To the best of our knowledge, and in conclusion, this is a pioneering study to show that the combination of berberine with the effective chemotherapeutic agent cisplatin exhibits an additive effect on growth inhibition in cancer cell lines. Future studies may further substantiate this and become promising strategies for the application of combination therapies in in vitro/in vivo trials.

## Figures and Tables

**Table 1:** Effect of *Berberis vulgaris* root extract (*Bv*RE) on cisplatin-induced hepatotoxicity and on liver tissue oxidative status. Serum alanine transaminase (ALT) and serum aspartate aminotransferase (AST) levels were measured in units per liter (U/L), while liver tissue malondialdehyde (MDA) and catalase (CAT) were measured in nmol/10 mg and U/mg of protein, respectively.

Groups	ALT (U/L)	AST (U/L)	MDA (nmol/10 mg)	CAT (U/mg)
Normal control group	34.00 ± 4.73	40.60 ± 2.70	22.5 ± 0.8	25.98 ± 4.30
Prophylactic control group	109.00 ± 5.85 ^a^	76.80 ± 6.34 ^a^	38.5 ± 1.54 ^a^	14.84 ± 2.02 ^a^
Prophylactic group	35.20 ± 4.65 ^a,b^	45.80 ± 4.17 ^a,b^	16.5 ± 1.45 ^a,b^	25.68 ± 2.96 ^a,b^
Curative control group	102.81 ± 4.71 ^a^	77.20 ± 6.65 ^a^	31.5 ± 1.33 ^a^	17.18 ± 1.98 ^a^
Curative group	44.40 ± 5.17 ^a,c^	61.00 ± 6.13 ^a,c^	18.8 ± 0.98 ^a,c^	18.78 ± 3.56 ^a,c^

The values of hepatic function and oxidative status parameters are expressed as the mean ± S.D. of five observations (*n* = 5), where ^a^ represents the comparison with the normal control as *p* < 0.05, ^b^ represents the comparison with the prophylactic control as *p* < 0.05, and ^c^ represents the comparison with the curative control as *p* < 0.05 (one-way ANOVA followed by a post-hoc Scheffé test).

**Table 2:** Effect of *Berberis vulgaris* root extract (*Bv*RE) on cisplatin-induced nephrotoxicity and kidney tissue oxidative status. Serum creatinine and urea levels were measured in mg/dL, serum total protein (TP) in gm/dL, kidney tissue malondialdehyde (MDA) level in nmol/mg protein, and kidney tissue catalase (CAT) in U/mg protein.

Groups	Creatinine (mg/dL)	Urea (mg/dL)	MDA (nmol/mg)	CAT (U/mg)	TP (gm/dL)
Normal control group	0.82 ± 0.14	31.40 ± 0.95	0.62 ± 0.16	20.44 ± 1.95	6.55 ± 0.51
Prophylactic control group	3.34 ± 0.45 ^a^	37.20 ± 1.01 ^a^	2.50 ± 0.58 ^a^	3.96 ± 1.07 ^a^	3.28 ± 0.66 ^a^
Prophylactic group	0.94 ± 0.27 ^a,b^	76.80 ± 1.13 ^a,b^	1.33 ± 0.20 ^a,b^	11.18 ± 1.36 ^a,b^	5.13 ± 0.87 ^a,b^
Curative control group	3.08 ± 0.51 ^a,c^	45.80 ± 0.81 ^a^	1.67 ± 0.36 ^a^	7.09 ± 1.10 ^a^	3.69 ± 0.66 ^a^
Curative group	1.10 ± 0.38 ^a,c^	77.20 ± 0.96 ^a,c^	1.00 ± 0.17 ^a,c^	12.36 ± 1.19 ^a,c^	4.03 ± 0.59 ^a,c^

The values of renal function and oxidative status parameters are expressed as the mean ± S.D. of five observations (*n* = 5), where ^a^ represents the comparison with the normal control as *p* < 0.05, ^b^ represents the comparison with the prophylactic control as *p* < 0.05, and ^c^ represents the comparison with the curative control as *p* < 0.05 (one-way ANOVA followed by a post-hoc Scheffé test).

**Table 3:** Effect of *Berberis vulgaris* root extract (*Bv*RE) on cisplatin-induced hyperlipidemia. Serum triglycerides (TG) and total cholesterol (TC) were measured in mg/dL.

Groups	TG (mg/dL)	TC (mg/dL)
Normal control group	160.80 ± 5.38	63.00 ± 8.00
Prophylactic control group	243.80 ± 4.90 ^a^	89.00 ± 11.46 ^a^
Prophylactic group	222.40 ± 9.80 ^a,b^	58.40 ± 6.98 ^a,b^
Curative control group	236.60 ± 10.27 ^a^	82.00 ± 7.28 ^a^
Curative group	203.60 ± 9.08 ^a,c^	63.20 ± 9.37 ^a,c^

The values of serum lipid parameters are expressed as the mean ± S.D. of five observations (*n* = 5), where ^a^ represents the comparison with the normal control as *p* < 0.05, ^b^ represents the comparison with the prophylactic control as *p* < 0.05, and ^c^ represents the comparison with the curative control as *p* < 0.05 (one-way ANOVA followed by a post-hoc Scheffé test).

**Table 4:** Dose regimen of cisplatin and *Bv*RE.

Group	Diet	Sacrificed
Control group	Water and normal diet along with administration of normal saline (i.p.)	12th day
Prophylactic control group	Distilled water the 1st–8th day followed by a single dose of cisplatin (4 mg/kg) on the 9th day	12th day
Prophylactic group	*Bv*RE (500 mg/kg/day) from the 1st–8th day followed by a single dose of cisplatin (4 mg/kg) on the 9th day	12th day
Curative control group	Animals administrated with a single dose of cisplatin (4 mg/kg) on the 1st day	12th day
Curative group	Single dose of cisplatin (4 mg/kg) on the 1st day followed by *Bv*RE (500 mg/kg/day) from the 4th–11th day	12th day
